# Core components of a rehabilitation program in pediatric cardiac disease

**DOI:** 10.3389/fped.2023.1104794

**Published:** 2023-05-31

**Authors:** Ana Ubeda Tikkanen, Joshua Vova, Lainie Holman, Maddie Chrisman, Kristin Clarkson, Rachel Santiago, Lisa Schonberger, Kelsey White, Daryaneh Badaly, Naomi Gauthier, Tam Dan N. Pham, Jolie J. Britt, Scott E. Crouter, Maeve Giangregorio, Meena Nathan, Unoma O. Akamagwuna

**Affiliations:** ^1^ Department of Pediatric Rehabilitation, Spaulding Rehabilitation Hospital, Boston, MA, United States; ^2^ Department of Cardiac Surgery, Boston Children’s Hospital, Boston, MA, United States; ^3^ Department of Orthopedic Surgery, Boston Children’s Hospital, Boston, MA, United States; ^4^ Department of Physical Medicine and Rehabilitation, Harvard Medical School, Boston, MA, United States; ^5^ Department of Physiatry, Children’s Healthcare of Atlanta, Atlanta, GA, United States; ^6^ Department Pediatric Physical Medicine and Rehabilitation, Cleveland Clinic, Cleveland, OH, United States; ^7^ Wolff Center, University of Pittsburgh Medical Center, Pittsburgh, PA, United States; ^8^ Department of Pediatric Physical Medicine and Rehabilitation, UPMC Children's Hospital of Pittsburgh, Pittsburgh, PA, United States; ^9^ Department of Otolaryngology and Communication Enhancement, Boston Children’s Hospital, Boston, MA, United States; ^10^ Learning and Development Center, Child Mind Institute, New York, NY, United States; ^11^ Department of Cardiology, Boston Children’s Hospital, Boston, MA, United States; ^12^ Department of Pediatric Cardiology, Baylor College of Medicine and Texas Children's Hospital, Houston, TX, United States; ^13^ Department of Kinesiology, Recreation, and Sport Studies, The University of Tennessee Knoxville, Knoxville, IL, United States; ^14^ Department Pediatric Physical Medicine and Rehabilitation, Texas Children's Hospital, Houston, TX, United States; ^15^ Department of Physical Medicine and Rehabilitation, Baylor College of Medicine, TX, United States

**Keywords:** children, heart, rehabilitation, function, cardiac

## Abstract

There is increasing effort in both the inpatient and outpatient setting to improve care, function, and quality of life for children with congenital heart disease, and to decrease complications. As the mortality rates of surgical procedures for congenital heart disease decrease, improvement in perioperative morbidity and quality of life have become key metrics of quality of care. Quality of life and function in patients with congenital heart disease can be affected by multiple factors: the underlying heart condition, cardiac surgery, complications, and medical treatment. Some of the functional areas affected are motor abilities, exercise capacity, feeding, speech, cognition, and psychosocial adjustment. Rehabilitation interventions aim to enhance and restore functional ability and quality of life for those with physical impairments or disabilities. Interventions such as exercise training have been extensively evaluated in adults with acquired heart disease, and rehabilitation interventions for pediatric patients with congenital heart disease have similar potential to improve perioperative morbidity and quality of life. However, literature regarding the pediatric population is limited. We have gathered a multidisciplinary team of experts from major institutions to create evidence- and practice-based guidelines for pediatric cardiac rehabilitation programs in both inpatient and outpatient settings. To improve the quality of life of pediatric patients with congenital heart disease, we propose the use of individualized multidisciplinary rehabilitation programs that include: medical management; neuropsychology; nursing care; rehabilitation equipment; physical, occupational, speech, and feeding therapies; and exercise training.

## 1. Introduction

In pediatric patients with congenital heart disease (CHD), postcardiac surgery morbidity and mortality have decreased over the past decades. In the United States, survival in the first year of life for children with critical CHD (i.e., those for whom surgery or intervention is needed) is 75.2% vs. 97.1% for those with non-critical CHD (1). However, a growing body of research has found that some pediatric patients with CHD experience long-term functional impairment and some degree of disability following surgery, particularly concerning functional outcomes (2, 3), neurodevelopment (3, 4), and exercise capacity (5, 6).

There are only a few pediatric cardiac rehabilitation programs and there is little published research about their effectiveness. Recent research showed that almost half of patients that undergo surgery for CHD required some sort of rehabilitation therapy in the acute postoperative period (2). In contrast, in adults with acquired heart disease, the benefits of post-surgical cardiac rehabilitation have been widely proven. However, only 10%–20% of those adult patients who would benefit from this type of intervention participate in a rehabilitation program (7).

Rehabilitation management of pediatric cardiac patients frequently starts in the inpatient acute setting, especially for children with severe cardiac defects who spend a significant amount of time in the hospital, and it continues in the outpatient setting (2). Of those pediatric programs documented in the literature, most use a team format, and they include inpatient acute care programs, home-based programs, and outpatient-based programs. The outpatient exercise training programs have demonstrated the safety and feasibility of their particular models of management, and they measure patient improvement using outcome metrics such as maximal oxygen consumption, exercise tolerance, and quality of life (8).

We offer these definitions for the reader's ease:

“Neurodevelopment” refers to the neurologic and developmental trajectory of a child.
“Functional outcomes” refer to the neurodevelopmental trajectory of children as well as their reported re-integration into their communities, ability to gain independence, and overall quality of life.
“Function” is a key tenet in rehabilitation and encompasses all aspects of daily life, including mobility, communication, feeding, and other activities of daily living.
“Rehabilitation management” includes an interdisciplinary approach to improving function as defined previously. We include key components of this rehabilitation team further in this paper.

**Figure 1:**
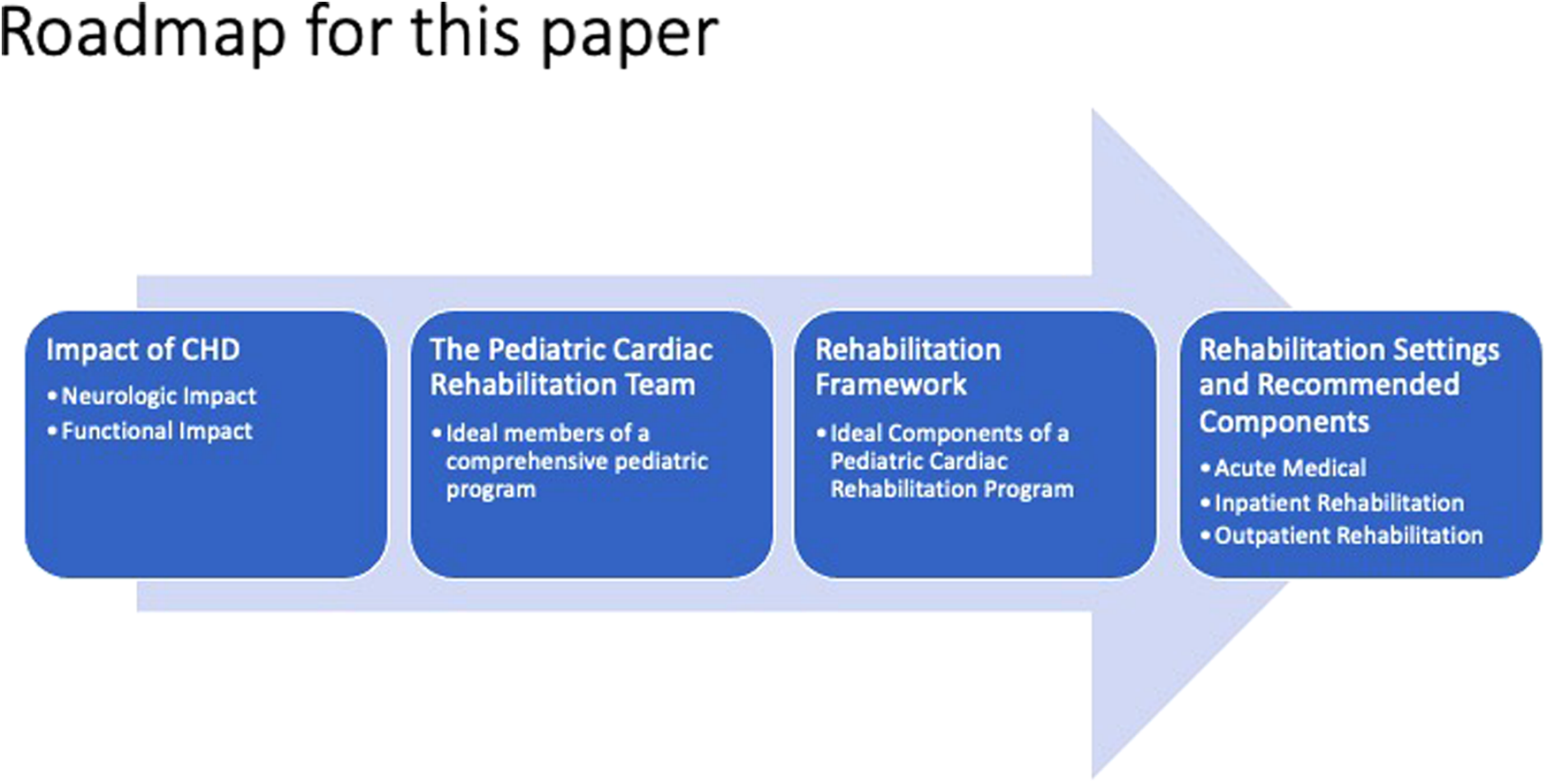
Roadmap.

In this paper, we briefly discuss the functional deficits in CHD and then explore the potential role of each member of the multidisciplinary pediatric cardiac rehabilitation team in addressing them (Table 1). We then propose a framework for multidisciplinary pediatric cardiac rehabilitation in both inpatient and outpatient settings (Figure 1), and we highlight the unique considerations of such programs for pediatric CHD vs. those addressing adult-acquired heart disease.

**Table 1:** Roles of the multidisciplinary team.

Team member	Role
Physical medicine and rehabilitation Physicians (Pediatric Physiatrists)	• Specialize in rehabilitation care aimed at promoting functional recovery, age-appropriate independence, optimal neurodevelopmental outcomes and maximizing quality of life. • Providers develop treatment plans, recommend medical interventions as appropriate. • Work in conjunction with therapies, including physical occupational and speech/language therapy, towards functional goals. • Provide education, coordinate care and prescribe rehabilitation equipment (e.g., braces, walkers).
Cardiologists/cardiovascular surgeons	• Provide a cardiac evaluation and clear the patient for rehabilitation. • Help assess the exercise capacity and design the exercise training piece. • Provide guidance as to specific precautions for the patient depending on the type of surgical intervention, the underlying cardiac physiology and rhythm disorders.
Nursing	• Specialize in the delivery of skilled nursing care (complex dressings, IV medications). • Provide cardiac assessments and data collection (vital signs, weights, I/O). • Provide in-depth education (symptom recognition and management, medications). • Assist with coordination of all patient care among the multidisciplinary team. • Assess overall health status, tolerance to all therapies, and support activities of daily living. • Ensure patient/family understanding and compliance with all aspects of care.
Exercise physiologist	• Play a role in monitoring and administering exercise regimens including electrocardiographic monitoring, expiratory gas analysis. • Work in tandem with the team to determine the best fitness program for the patients.
Speech-language Pathologists	• Specialize in care focusing on the evaluation and treatment of speech, language, feeding, and swallowing. • Assist in the recovery of age-appropriate cognition, arousal, speech skills, expressive and receptive language, pragmatics and social skills, reading ability, as well as feeding and swallowing • Promote functional communication with providers and loved ones including potential implementation of augmentative and alternative communication (AAC).
Physical therapists	• Provide clinical applications in the restoration, maintenance, and promotion of optimal physical function, wellness, fitness, and quality of life as it relates to movement and overall health. • Help prevent the onset, symptoms, and progression of impairments, functional limitations, and disabilities that may result secondary to cardiac dysfunction.
Occupational Therapists	• Work to restore functional skills and activities of daily living, enabling children to be independent within their home, school, and community settings. • Use restoration and compensatory strategies to promote recovery from a cardiac condition. • Help children and families understand how to manage and improve cardiac health within their daily schedules and routines through training and education.
Psychologists and neuropsychologists	• Specialize in the evaluation and treatment of a broad array of neurodevelopmental, neurocognitive, and psychiatric conditions. • Conduct developmental and neuropsychological evaluations to help with treatment planning, implementation of supports in school, promoting quality of life, among other goals. • Provide psychoeducation and psychotherapy for children and their families struggling with adjustment concerns related to medical condition or comorbid psychiatric conditions, as well as provider cognitive rehabilitation and academic remediation.

## 2. Our goal

Our goal is to provide a framework for developing a multidisciplinary rehabilitation program to improve long-term function and quality of life for pediatric patients with CHD. In the course of this paper we will:

• Increase awareness of specific functional complications related to CHD
• Provide a rehabilitation framework for pediatric patients with CHD
• Offer recommendations to decrease the functional impact of CHD surgery and associated complications
• Provide recommendations to improve the quality of life in pediatric patients with CHD
• Give guidance on educating parents and patients to help decrease anxiety regarding physical activity
• Promote a cardio-healthy lifestyle with physical activity and an appropriate heart-healthy diet, decrease CV risk factors, and increase medication compliance
• Demonstrate how to provide functional support throughout childhood and into adulthood
• Provide guidance on helping pediatric patients achieve maximum independence

**Figure 2:**
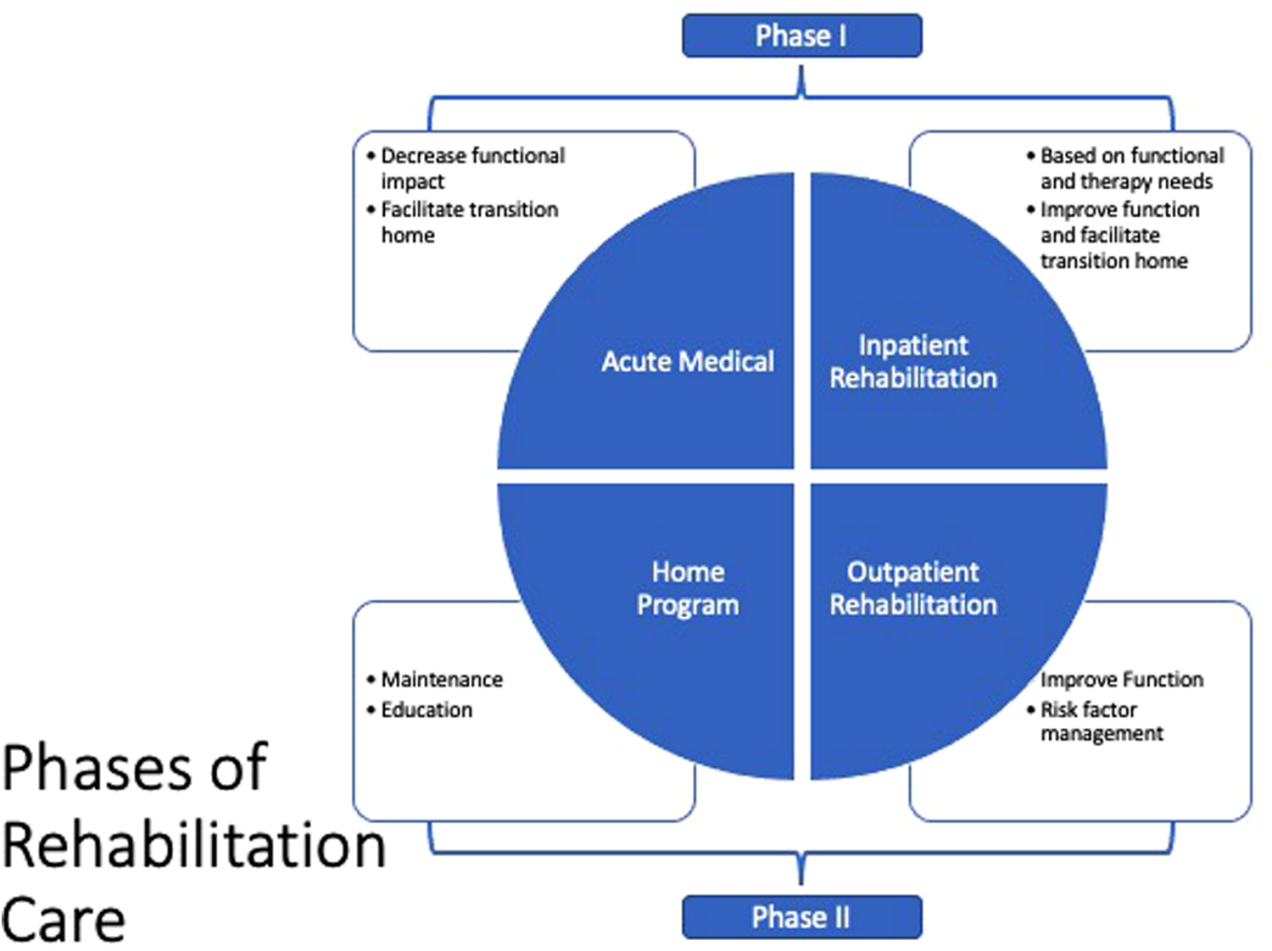
Rehabilitation settings.

We believe there are two essential time points for providing rehabilitation care for pediatric patients with CHD (Figure 2).

Acute post-surgery (Phase I): In this period, the aim is to decrease the acute functional impact of the surgical intervention and possible post-surgical complications, and facilitate the transition home. Acute post-surgery care can take place in the acute medical setting, acute inpatient rehabilitation or a long-term acute care setting. This phase is more relevant in CHD vs. adults with acquired heart disease.
Post-surgery (Phase II): This subacute phase, which is analogous to the same phase in acquired heart disease, includes exercise training for older pediatric patients and cardiovascular risk factor management adapted to the functional needs of the pediatric patient population. Additional outpatient rehabilitation therapy services may also be needed.

## 3. Functional deficits in CHD

### 3.1. Neurologic

Children born with complex CHD have smaller and more immature brains at birth compared to children without CHD (9). This brain immaturity could increase the risk of periventricular leukomalacia (PVL), which is associated with cerebral palsy, after cardiac surgery. The most common cause of stroke in children is CHD itself or the surgery for CHD. Additionally, brain injury affects 55% of neonates in the peri-cardiac surgery period, with most of these cases being PVL (10). In patients who require extracorporeal membrane oxygenation (ECMO) support, stroke incidence is higher—around 12.3% (11). For patients who required a ventricular assist device (VAD), the incidence of having at least one stroke is 29% (12). This neurologic injury makes them susceptible to having some form of motor, speech, cognitive, or feeding disability, which in turn affects their activities of daily living.

Longer-term abnormal neurodevelopment outcomes have been extensively described in patients with CHD (3, 13). These deficits are varied, affecting areas of functioning such as language (14), executive function (15), and visual processing (16) skills, and they can adversely affect school performance and quality of life. Multiple risk factors have been identified, including chronic cyanosis, genetic and syndromic abnormalities, medical and surgical therapies, brain injury, comorbidities, and a lack of exposure to normal developmental stimuli in the intensive care unit (ICU) (13).

As more neurodevelopmental follow-up programs are established and refined, neurologists, psychologists, neuropsychologists, and other health providers have outlined their experiences and recommendations for the care of children with congenital heartdefects (17, 18). In addition, the American Heart Associationandthe American Academy of Pediatrics have published their respective guidelines for the evaluation and management of developmental and neuropsychological outcomes among children with CHD (13). Most recently, the Cardiac Neurodevelopmental Outcome Collaborative provided guidance on developmental evaluations from birth through age 5 and on neuropsychological evaluations for school-age children (18, 19).Although these collective recommendations and guidelines arecritical in directingthe neurodevelopmental care of childrenwith CHD, theydo not specifically address the functionalneeds of CHD patients. Our framework attempts to fill this critical gap.

### 3.2. Feeding

Feeding difficulties are not uncommon in patients with CHD (20). Up to 50% of neonates with complex CHD require tube feeding at discharge following cardiac surgery (21). These feeding disorders can persist over time in over 20% of these patients (22). The etiology of feeding difficulties is thought to be multifactorial: abnormal development of the operculum in the brain of patients with complex CHD that is related to feeding and speech difficulties (9); decreased intake or increased energetic expenditure in heart failure (23); brain injury (24); vocal cord dysfunction (25); suck-swallow-breathe discoordination; laryngopharyngeal dysfunction (26); and reflux or oral aversion after prolonged intubation. Feeding difficulties can lead to complications such as aspirations (27), prolonged hospitalization, and, importantly, an adverse effect on patients' growth and development (28).

### 3.3. Speech and language

In CHD, speech and language difficulties include delays and/or impairments in the motor and cognitive aspects of communicative functioning (29–31), which can range in severity from mild to severe. Deficits in speech production, including articulation or phonological disorders, motor speech disorders, voice and/or resonance disorders, and dysfluencies may also be present (32). Patients are at risk of receptive and/or expressive language difficulties, as well as challenges in social pragmatics that may be part of autism spectrum disorder or in isolation (29, 33). Finally, it is not uncommon in this patient population to observe language-based learning disabilities and deficits in attention and executive functioning, which can also impact communicative functioning (34).

### 3.4. Exercise capacity

An active lifestyle plays a critical role in health outcomes. As noted above, neurological and cognitive impairments can also impact exercise capacity and physical activity. Thus, it is important to consider all aspects of health-related fitness. This is important because exercise and physical activity are two different constructs (the former is a subcomponent of the latter) and each can result in different outcomes, all of which are important to long-term health in complex CHD.

Patients with complex CHD tend to have lower median oxygen consumption (peak VO_2_), the gold standard measurement of peak aerobic exercise capacity (35), which in this patient population is related to morbidity and mortality (6, 36). A number of studies have shown that decreased exercise capacity can improve with physical activity in patients with CHD (37). Limited literature is available at this time on pediatric CHD patients and improvement in outcomes. However, recent data suggests that exercise training in children with CHD may improve exercise parameters as well as quality of life, without serious adverse outcomes (38, 39). In addition to peak VO_2_, it is important to understand other health-related components of a patient's fitness, including movement deficits, muscular fitness, barriers to physical activity, and physical activity preferences.

## 4. The rehabilitation framework

We believe rehabilitation intervention in CHD patients should be a multidisciplinary team effort. We describe the potential team members and their roles in Table 1. While the roles are described as independent, there is significant overlap between different professionals in both assessments and interventions. Different functional impairments might be addressed in multiple rehabilitation therapy domains, such as deconditioning or weakness treated with both physical therapy and exercise training.

### 4.1. Inpatient rehabilitation in CHD (Phase I)

Inpatient rehabilitation generally takes place in the hospital setting after cardiac surgery or hospitalization for heart failure. With guidance from the cardiology and cardiovascular surgery team, plans for therapeutic rehabilitation interventions should be developed and initiated as soon as possible to optimize mobility and prevent complications during the post-CHD and -transplant surgical course. As most acute medical inpatient settings have limited physical and occupational therapy resources, inpatient rehabilitation management frequently falls to the bedside nursing staff. Nurses are also among the first care team members to interact with and assess post-operative patients; in the ICU, early assessment and recognition of neurological, vocal, feeding, and motor abilities begin immediately after surgery thus increasing the opportunity for more timely rehabilitation interventions.

While monitoring the patient's tolerance to aerobic and therapeutic exercise, physiatrists can ensure therapeutic activities are aligned with the patient's goals, medical status, and function. During the hospital course, the physiatrist will assess and determine the appropriate level of rehabilitation services required after discharge, such as inpatient rehabilitation, an intensive day rehabilitation program, or an outpatient rehabilitation plan. Maintenance of safe activity is critical at this juncture to mitigate the effects of debility known to occur in the acute inpatient setting. In the pediatric population, minimal adverse outcomes have been reported with early mobilization programs and they are often used by bedside nursing (40).

There is already a precedent for inpatient pediatric rehabilitation for patients with heart failure and for those with ventricular assist devices (8). Patients in these programs have, on average, three to five sessions of therapy per week, with no adverse effects reported. With this existing framework in mind, we make the following proposals for implementing a pediatric rehabilitation program in the inpatient setting.

#### 4.1.1. Nursing

Nurses are unique in that they are among the first team members to assess patients' functional health and they also spend the most direct care time with patients. Nursing assessments, therefore, provide early and rapid evaluation of potential threats to functional health, allowing for more timely communication with the overseeing provider and necessary specialists. Nursing is also able to institute supportive interventions for vocal, feeding, and motor disabilities before a patient is deemed ready for formal physical and occupational therapies. Once a patient is screened by physiatry and the rehabilitation therapists, nursing plays an essential role in ensuring therapy recommendations are tolerated by the patient and, if so, followed. Nursing also plays a large role in patient education, including but not limited to medication management, symptom awareness and management, and discharge readiness, all of which are specific to each patient. When transfer to an inpatient rehabilitation facility is likely, nursing plays a significant role in guiding and preparing the patient and family for that transition.

#### 4.1.2. Physical and occupational therapy

Physical and occupational therapy plays a vital role in caring for pediatric patients who are hospitalized for CHD. These patients are at increased risk of prolonged immobility, which often results in long-term sequelae that can be best supported through a wide range of therapeutic activities. These activities begin with early mobilization, i.e., a patient's active participation in therapeutic activity within 48–72 h of admission, upon hemodynamic stability, or when the medical team deems it appropriate (40, 41).

The goals of care for physical therapy and occupational therapy are to maximize functional mobility and independence while minimizing the deleterious effects of bedrest and to prevent deconditioning; to assess and facilitate achievement of developmental milestones and to help provide appropriate recommendations for follow-up services upon discharge.

The child should be examined and evaluated for physical and occupational therapy, with individualized interventions (Tables 2–4) recommended that are neurodevelopmentally appropriate and specific to the child's functional deficits.

**Table 2:** Clinical assessment and education considerations for physical and occupational therapy.

Review of the electronic medical record and patient/family interview
• Respiratory and cardiac support at time of physical therapy session. • Patient appropriateness for therapy is determined in conjunction with the medical team. There are respiratory and cardiac parameters for early mobilization in adults although these have not been determined in the pediatric population (42). • Obtain functional and or neurodevelopmental history through chart review and parent report • Review therapy history, home environment, current equipment, and orthosis use
**Parent and patient education**
• Sternal precautions (43) as they relate to functional or developmental activity • Many practices start prone positioning 2 weeks post-surgery • Importance of progressing towards baseline functional activity • Educate patient and family on diagnosis and recovery process including • Energy management and pacing, self-monitoring techniques, and activity progression

**Table 3:** Assessment and intervention considerations for physical therapy (as applicable, assessment and intervention should be patient specific and not all of the following variables may be included depending on patients age, cognitive level, needs, etc.).

Assessment domain	Assessment considerations	Intervention considerations
Aerobic capacity/endurance	• Six-minute walk test: Assesses distance walked over 6 min as a sub-maximal test of aerobic capacity/endurance • Assess HR, BP, RPE (rate of perceived exertion) and METS, O_2_ sat if applicable • Assess RPP (rate pressure product), as this can standardize intensity across different modes of activity • Telemetry monitoring if deemed necessary by cardiology team.	
Mobility	• Current Level of Mobility • Use of scales such as Test of Gross Motor Development (TGMD-3) (44) • Dynamic Gait Index: Assesses individual's ability to modify balance while walking in the presence of external demands	• Gait Training • Depending on current functional state, therapeutic goals start with bed mobility, supine > sit > stand > progressive ambulation • Transfer training as necessary
Musculoskeletal screen	• Assess posture, scar location and mobility, and breathing patterns • Manual Muscle/Functional Strength Testing: Assesses muscle strength and function • Range of Motion: Assesses a specific joints range of motion either passively or actively. Consider evaluating: Shoulder Flexion, Lateral Trunk Flexion, Popliteal Angle, Ankle Dorsiflexion with knee extended	• Postural exercises • Breathing Mechanics • Initiation of Scar Tissue Mobilization • Strengthening with progression to light resistance as appropriate • Stretching • May include passive, active, or active-assisted range of motion

HR heart rate, BP blood pressure, RPE (rate of perceived exertion) and METS metabolic equivalents, O_2_ sat oxygen saturation.

**Table 4:** Assessment and intervention considerations for occupational therapy (as applicable, assessment and intervention should be patient specific and not all of the following variables may be included depending on patients age, cognitive level, needs, etc.)

Assessment domain	Assessment considerations	Intervention considerations
Activities of daily living	• Assess current level of independence in age-appropriate ADLs • Childhood Occupational Self-Assessment Scale	• Facilitate completion of ADLs and daily occupations in sitting > standing > functional mobility with item retrieval • Employ adaptive techniques for ADLs & independent ADLs if upper extremity discomfort or pain is present • Provide activity log worksheet to track participation in daily ADLs/occupations and RPE
Aerobic capacity/endurance	• Assess HR, BP, RPE and METS, O_2_ sat if applicable	• Challenge the patient's cardiovascular system by using occupations and a graded MET level approach • Instruct patient in use of appropriate Rating of Perceived Exertion (RPE) scale—i.e. Borg, OMNI • Understand how RPP relates to RPE for different modes of activity
Musculoskeletal screen	• Assess posture, scar location and mobility, and breathing patterns • Manual Muscle/Functional Strength Testing: Assessed muscle strength and function ○ Grip Strength ○ Tip Pinch ○ Lateral key pinch ○ Tripod pinch • Range of Motion: Assesses a specific joints range of motion either passively or actively. Consider evaluating: Shoulder Flexion, Elbow flexion/ extension, Wrist flexion/extension, finger flexion.	• Postural exercises • Breathing Mechanics • Initiation of Scar Tissue Mobilization • Strengthening with progression to light resistance as appropriate • Stretching • May include passive, active, or active-assisted ROM

ADLs activity of daily living, HR heart rate, BP blood pressure, RPP rate pressure product, RPE (rate of perceived exertion) and METS metabolic equivalents, O_2_ sat oxygen saturation.

#### 4.1.3. Speech and language therapy

Children with CHD are vulnerable to speech and language delays (29–31) which may warrant consultation with speech and language pathologist (SLP) services. These include baseline communication challenges, acute changes (e.g., due to stroke, vocal cord paralysis, ventilation requirements, etc.), or the effects of prolonged hospitalization. Early consultation may facilitate ongoing developmental support, communication access and patient-provider communication, and maintenance of skills. Table 5 outlines additional considerations for inpatient assessment of, and interventions for, speech, language, and communication needs in children with CHD.

**Table 5:** Assessment and intervention considerations for speech, language, and communication: (as applicable, assessment and intervention should be patient specific and not all of the following variables may be included depending on patients age, cognitive level, needs, etc.).

Assessment domain	Assessment considerations	Intervention considerations
Bedside environment	• Lighting • Noise • Impact of mounting equipment at bedside • Presence of medical devices and equipment • Staff access to patient • Storage of AAC tools at bedside	• Ensure adequate environment to promote therapy participation and bedside interactions
Cognition	• Sedation level • Ability to maintain wakefulness • Baseline/pre-morbid status • Medication effect • Sleep hygiene • Delirium • Attention to task • Attention to others • Symbolic understanding of visuals (e.g., photographs, picture-communication symbols, written text) • Memory	• Adjust task and length of session based on wakefulness, sedation, and ability to attend to task. • Conduct intervention when patient is expected to be most alert depending on medication schedule • Establish attention: reduce background noise, keep lights on, reduce distractions • Provide intervention tools and augmentative and alternative communication (AAC) strategies that incorporate the patient's current level of symbolic need. This may include use of pictures to represent language or concepts, written text, objects, and other symbols. • Promote use of memory aids and strategies to increase short and long term memory formation and recall. • Promote use of strategies to prevent and reduce delirium.
Sensory profile	• Vision: current and pre-morbid status ○ Availability of visual aids • Hearing: current and pre-morbid status ○ Availability of hearing aids, cochlear implant, or amplification equipment • Feasibility of wearing and tolerating use of sensory aids • Impact or interference of medical equipment • Impact or interference of swelling and incision sites • Sensory integration needs	• Ensure access to sensory aids including glasses, hearing aids, cochlear implants, etc. • Provide access to supplemental sensory aids as indicated (e.g. amplification systems, magnifying glass, etc.) • Integrate strategies to address sensory integration needs.
Expressive communication	• Primary language • Baseline/pre-morbid status • Expressive language skills • Speech intelligibility • Impact of respiration on speech and communication: • Impact of non-invasive ventilation on breath support, volume, articulation, and resonance (e.g. nasal cannula, HFNC, BiPap, CPap) ○ Speaking volume ○ Presence of endotracheal tube ○ Presence of airway anomalies that affect speech production ○ Tracheostomy cuff status ○ Speaking valve tolerance ○ Ventilator settings	• Ensure access to medically certified interpreters. Presented materials should be in the patient's primary language. As indicated, AAC tools should incorporate bi-direction and bilingual text or voice-output. • Provide intervention targeting changes in speech intelligibility to support functional communication and early rehabilitation. • Provide intervention targeting changes in expressive language skills to support functional communication and early rehabilitation. • Provide access to communication strategies to supplement oral speech that is not functionally intelligible. • Implement voice amplification to enhance speaking volume in patients with vocal cord paresis or paralysis. Refer to Otolaryngology as indicated. • Implement strategies to enhance breath support including but not limited to posture and positioning, pacing, and intermittent breaths.
Receptive communication	• Primary language • Baseline/pre-morbid status • Ability to follow verbal directions • Ability to answer yes/no questions • Ability to comprehend complex messages • Ability to comprehend gestures and physical behaviors *(Patient and family's level of health literacy and prior healthcare experiences should be considered during all medical discussions)*	• Ensure access to medically certified interpreters. Presented materials should be in the patient's primary language, including those to support augmented input. As indicated, AAC tools should incorporate bi-direction and bilingual text or voice-output. • Provide intervention targeting changes in receptive language to support functional communication and early rehabilitation. • Provide AAC strategies to support the child's ability to answer yes/no questions. This may include communication boards, voice-output communication aids, and other tools. Typically, access to “yes,” “no,” and “I don’t know” messages is encouraged to avoid limited answers to binary choices. • Provide augmented input and supplemental visuals as indicated to support comprehension. This may include visual schedules, First-Then schedule, Social Stories™, or visual scene displays
Literacy	• Comprehension of written words • Ability to spell single words, phrases, and sentences. • Ability to use a keyboard • Speed of access to various keyboard layouts	• For literate patients, or those with emerging literacy, provide intervention that targets encoding and decoding at developmentally appropriate levels. • For non-speaking patients, incorporate AAC strategies that include text-to-speech or use of letter boards to promote production of generative messages.
Physical communication access	• Fine and gross motor skills • Use of gestures for functional communication • Use of facial expression for functional communication • Use of eye gaze, eye blinks, and eye pointing • Motor control and coordination • Ability to directly select icons on various displays (e.g. *via* pointing with hand, eyes, pointer, or other direct methods) • Ability to indirectly select icons on various displays (e.g. *via* switch scanning, partner-assisted scanning of messages, or other indirect methods) • Ability to write • Ability to draw • Need for mounting equipment to optimize access to AAC tools • Impact of medical devices and equipment (e.g. IV boards, restraints, EEG leads, chest physical therapy vests, etc.) • Positioning restrictions	• Tailor intervention strategies and tasks to accommodate for baseline physical skills and potential changes in physical access. • If unable to point directly to targets, incorporate alternative access methods including, but not limited to, eye gaze, partner-assisted scanning, and scanning with a switch (for high-technology AAC systems). • If unable to access the standard nurse-call system, provide an adapted nurse-call switch. • Work with OT and PT to determine the best access method and alternative strategies to promote participation in therapy and access to tools. This may include mounts, wedges, splints, styli or other pointers, and supportive seating solutions. • For non-speaking patients, incorporate the best access method to support independent selection of messages for functional communication.
Vocabulary selection	• Patient needs • Patient desires • Patient personality • Patient interests • Participation in play • Participation in medical discussions • Participation in social interactions • Ability to inquire and ask questions • Ability to opt out, decline, or protest	• Incorporate developmentally appropriate and patient-relevant vocabulary into speech-language therapy and AAC systems.

#### 4.1.4. Augmentative and alternative communication (AAC)

If a patient presents as non-speaking or with functional communication difficulty (e.g., due to mechanical ventilation), the use of AAC strategies may be required to establish reliable communication during hospitalization (45). The SLP should conduct a feature-matched assessment by matching the patient's strengths, skills, and needs to available tools and strategies (46), which may include a variety of no-tech, low-tech (e.g., communication boards, writing tools), and high-technology (e.g., speech-generating devices). If the child has receptive language issues, communication partners can use strategies to supplement language input. It should be noted that AAC does not impede the development of spoken language (47, 48) and may in fact increase speech production (49).

#### 4.1.5. Feeding and swallowing

Regardless of cardiac anatomy, all patients with CHD are at risk for feeding difficulties (50), failure to thrive, and dysphagia (20). Feeding difficulties and dysphagia are thought to be related to many of the concomitant issues mentioned above. Many newborns are discharged home with feeding tubes, given the difficulty of transitioning to full oral feeds (51). Infants benefit from early feeding interventions pre-operatively, as well as post-operatively, to avoid the need for gastrostomy placement (52). Research has demonstrated that infants with feeding difficulties are at increased risk of feeding struggles that persist in childhood (50), and therefore early speech-language pathology involvement is imperative.

Feeding assessment and management both for the in- and outpatient setting are further detailed in Tables 6, 7.

**Table 6:** General assessment considerations for feeding.

Assessment considerations
Review of the electronic medical record
• Cardiac diagnosis • Comorbidities, particularly those that may increase risk for feeding difficulties • Medications, including those that increase risk for dysphagia and impact alertness • ECMO cannulation site (if applicable) • Length of intubation given increased risk for dysphagia associated with increased length of intubation (53)
Obtain feeding history through chart review and parent report
• Preoperative feeding information • Feeding modality ○ Oral feeding ▪ Breast feeding ▪ Bottle feeding ▪ Baby food ▪ Solids ○ Non-oral enteral nutrition (e.g. NGT, GT, etc.) • Parenteral nutrition • Oral stimulation experience and exposure • Previous feeding concerns (concerns for aspiration, GER, endurance, etc.)

NGT, nasogastric tube; GT, gastric tube.

**Table 7:** Feeding and swallowing Management in the pediatric acute in and outpatient care setting: Assessment considerations (as applicable, assessments should be patient specific and not all of the following variables may be included during assessment depending on the patient's age, cognitive level, risk assessment, etc.) the screening, assessment, and intervention should be patient-specific and account for neurological etiology (e.g. stroke), cognition (e.g. attention to task, sedation, etc.), sensory domains (e.g. vision and hearing), and age.

Assessment domain	Assessment considerations	Intervention considerations
• Assess pre-feeding readiness	○ Physical state and motor assessment ○ State regulation ○ Oral-motor behavior and reflexes	Pre-feeding intervention • Skin-to-skin • Colostrum oral care • Facilitate hands to mouth *via* midline positioning and flexion • Non-nutritive sucking skills with pacifier • Offering therapeutic breast milk or formula Feeding environment (infant) • Limit distractions and decrease visual and auditory simulation • Assist infant in reaching a calm and alert state • Hold infant during feeding unless medically contraindicated • Support appropriate body positioning during feeding (arms to midline, flexion, neutral head/neck, supported legs/feet). Utilize swaddle as needed
• Positioning	○ Upright, reclined, flat, elevated side-lying ○ Well-supported, swaddled	• Semi-reclined • Fully upright ○ Utilize horizontal milk flow with bottle parallel to floor • Elevated side-lying position • Infant is positioned fully on their side with ears, shoulders, and hips in alignment
• Assess feeding skills during an oral trial (Infants):	Assess feeding skills during an oral trial: • Latch to nipple on bottle or breast • Nutritive sucking coordination • Physiologic stability throughout feeding • Endurance • State regulation (including ability to remain awake and engaged) • Possible concerns for aspiration ▪ Coughing ▪ Choking ▪ Changes in vital signs ▪ Congestion ▪ Increased work of breathing • Stress cues ▪ Eyebrow raise ▪ Eyelid flutter ▪ Furrowed brow ▪ “Worried look” ▪ Pulling off from nipple ▪ Splayed fingers, arms, legs ▪ Pushing away nipple • Post feeding assessment ▪ Physiologic state ▪ Energy	Changes in nipple flow rate. • Refer to Pados (53), to determine differences between brands and nipple flow rates • Consider slower flow nipples for infants demonstrating difficulty with coordination of the suck-swallow-breathe sequence, physiologic stability, stress cues, and concerns for aspiration • Consider faster flow nipples for infants demonstrating reduced efficiency with high number of sucks per swallow Restrictions to reduce fatigue • Consider time restrictions, volume restrictions, or limiting frequency of offering • Co-regulated pacing (54) • Providing a break during feeding by either tilting the bottle down towards the floor the break the infants latch, or full removal of nipple from the infants’ mouth • This break is offered based off of infant cues during feeding Thickening liquids • Pending recommendations from instrumental assessments (e.g., MBSS or FEES) • Special considerations should be made in CHD population given poor gut perfusion and increased risk for NEC when considering thickening agent • Prior to implementing thickening, consult medical team to discuss options Parent education • Provide support to identify infant readiness cues and feeding cues • Educate caregivers regarding typical feeding difficulties and concerns • Provide guidance regarding when to discontinue oral feeding attempt
• Assess feeding skills during an oral trial (Child):	• Assess pre-feeding readiness • Assess feeding skills during an oral trial including: ○ Oral motor skills for drinking (bottle, straw, sippy cup, open cup, etc) ○ Oral motor skills for spoon feeding ○ Oral motor skills for solids ○ Physiologic stability throughout feeding ○ Endurance ○ State regulation (including ability to remain awake and engaged) ○ Possible concerns for aspiration ○ Sensory based difficulties ○ Refusal behaviors • Post feeding assessment ○ Physiologic state ○ Energy level • Parental interactions during meal times (not limited to): Parent stress level, bargaining/bribing, management of child's behaviors, positive/negative meal time language	Feeding environment • Limit distractions and decrease visual and auditory simulation (e.g., attempt to limit all screen time during meals) • Assist child in transitioning to table for meal • Support appropriate body positioning during feeding • Provide structured meal time schedule Parent education • Model use of positive meal time talk and provide education around reducing negative comments • Discourage bite-counting during meals • Avoid persistent feeding techniques (e.g. forcing bites in child's mouth) • Consider providing education around the “Division of Responsibility” during meal times (55) Oral motor impairments • Target oral motor movements to improve: • Cup drinking and straw drinking. May consider adaptive equipment as needed • Spoon feeding • Chewing and biting Sensory-based difficulties • Sensory challenges • Involve Occupational Therapy to target general sensory challenges • Consider utilizing techniques prior to meal times to improve sensory awareness prior to eating • Sensory desensitization with food • Use a graded system to slowly desensitize children to different aspects of non-preferred and novel foods • Consider utilizing Sequential-Oral-Sensory (SOS) Approach (56) • May implement Food Chaining strategies in therapy (57) Learned behavioral feeding difficulties • Consider consult with behavioral psychologist to provide further support • Target maladaptive feeding patterns and meal time interactions • Provide framing for children around their own feelings during meal times • Model strategies to improve positive behaviors and reduce negative behaviors Aspiration Pending recommendations from instrumental assessments (e.g. MBSS or FEES) • Consider positional strategies if able • Limit sip size by utilizing narrow diameter straws, slotted open cup, or single sip straws Thickened liquids (with medical team approval)

MBBS modified barium swallow study, FEES fiberoptic endoscopic evaluation of swallowing.

#### 4.1.6. Psychology and neuropsychology

##### 4.1.6.1. Psychological and neuropsychological screening

Children in ICUs and those in inpatient medical and rehabilitation units can benefit from systematic and focused psychological and neuropsychological consultation to: identify those with the highest risk of developmental, cognitive, and psychosocial concerns; aid with connecting patients and families with needed support; prepare children and families for the next stages of recovery; and provide guidance on additional neuropsychologic and developmental evaluation throughout recovery (58, 59). For more details see Table 8.

**Table 8:** Psychological and neuropsychological evaluation and intervention.

	Assessment considerations	Intervention considerations
Inpatient developmental care	• Consider the physical environment's impact on children's development and self-regulation. • Assess the caregiver relationships and understanding of cues. • Assess caregiver stress, mental health status, and resources.	• Support parents and their relationships with their child. • Train parents and providers in reading infant cues so as to respond to and address their individual needs.
Developmental and neuropsychological evaluations	• Developmental (or neurodevelopmental) evaluations typically refer to evaluations of early cognitive, language, motor, social, and adaptive skills among infants, toddlers, and young children who have yet to reach school age. Such evaluations may be performed or supervised by a psychologist, neuropsychologist, or other appropriately trained clinicians. • Neuropsychological evaluations, conducted or supervised by neuropsychologists, are comprehensive evaluations of cognitive abilities, academic skills as well as emotional, behavioral, social, and adaptive functioning, interpreted within the context of brain-behavior relationships. • Developmental or neuropsychological *screening* may be indicated a) to briefly screen functioning in intensive care units, b) at the start of inpatient rehabilitation to tailor treatment goals and interventions, c) at discharge from inpatient rehabilitation to guide transition planning for community and school supports, and d) shortly after discharge if not completed prior (∼1 month). • Patients with CHD referred to outpatient rehabilitation should complete developmental or neuropsychological *evaluations*, depending on their age, at the start and completion of their rehabilitation programs. Initial evaluations can help guide the rehabilitation team's goals and interventions; final evaluations should help guide transition planning. • Psychometrically-sound measures should be included within screenings along with a review of history and behavioral observations. • Brief screening for infants and toddlers: assesses the acquisition of early milestones, attention and regulation, and feeding and sleeping patterns (as needed in collaboration with other disciplines). • Further screening for infants and toddlers: assesses the above plus conduct developmental screening, which might include the Early Years Toolbox, Neonatal Behavioral Observation Scale or the Bayley Scales of Infant and Toddler Developmental Screening Test. • Comprehensive developmental evaluations for toddlers might assess cognitive, language, motor, adaptive, and self-regulatory functioning. As needed, evaluations may consider symptoms of autism spectrum disorder. • Screening for preschool and school-age children: assess the acquisition of preacademic or academic skills, pain, fatigue, traumatic stress, anxiety, and depression. • Further screening with school-age children: assess above plus conduct neuropsychological screening, which might focus on attention, processing speed, executive functioning, and psychosocial adjustment. If not assessed by speech and language therapy and occupational therapy, assess language abilities and visuomotor skills. • Comprehensive neuropsychological evaluations for preschool and school-age children might consider intellectual functioning, attention, processing speed, executive functioning, language abilities, visuomotor skills, learning and memory, preacademic or academic skills, and psychosocial adjustment. Among younger children, limited assessment may be possible in certain domains (e.g., executive functioning, memory). As needed, evaluations may consider social cognition and adaptive functioning. • The need for comprehensive evaluations should be determined based on risk documented within screening assessments, high risk medical events and conditions, standard of care for children with CHD, and goals. • Among infants and toddlers, factors affecting the evaluation might include reduced endurance, muscle tone, delayed language skills, and variable attention, among others. • Evaluations may need to be modified for children who have sensory or motor impairments, are nonverbal, are moderately to several intellectual disabled, and come from linguistic and cultural backgrounds that differ from the clinician.	• Oral and/or written feedback on the results of evaluations should be provided to patients (when developmentally and medically appropriate) and their families • Share findings with the rehabilitation team and other providers. • Consult with school personnel and/or participate in educational meetings as needed. • Feedback should include specific recommendations for early intervention services, academic accommodation and special education services including homebound instructional services, facilitating return to school, rehabilitation medicine assessment, rehabilitation therapies, as needed. • Make recommendations for mental health treatment and provide information on support groups for children and families. • Reinforce education on healthy habits going forward. • Help families build realistic expectations based on their child's abilities.
Cognitive Rehabilitation and Academic Remediation	• Consider the results of neuropsychological evaluations, evaluations from allied providers, and school records.	• Cognitive rehabilitation and academic remediation should be evidence-based and delivered by qualified providers trained in specific interventions. • School-age cardiac patients may particularly benefit from interventions for attention and executive functioning skills. • Research has outlined the potential for computer-based training programs for working memory among children with CHDs (60, 61) • Teaching children strategies for regulating their behavior (e.g., teaching children about executive functioning skills, practicing skills within academic tasks, teaching strategies for planning before acting) may be more beneficial than computer-based training. Examples of programs include Organizational Skills Training (62). • Although efforts should first be made to access academic remediation though children's school systems, there may be need for intervention above and beyond services in school. Children should access evidence-based interventions, which may be facilitated by appropriately trained educators, rehabilitation therapists, and psychologists.
Mental Health Care	• Infants and toddlers with CHD referred to rehabilitation should be assessed for attention and regulation, and feeding and sleeping patterns. • School-age patients should be assessed for psy­chological concerns, such as anxiety, depression, traumatic stress, disruptive behaviors, and social skills deficits, and should be provided with psychological in­terventions if abnormal findings are observed. • School-age CHD patients referred to rehabilitation should be assessed for barriers to treatment compliance and should be provided with psychological in­terventions if needed. • For children engaged in psychological intervention, psychometrically sound assessment tools should be repeated over the course of rehabilitation to ensure ongoing monitoring of symptoms and provide outcome measures of care.	• Psychological in­terventions should be evidence-based and delivered by licensed mental health professionals trained in specific interventions. • Motivational interviewing strategies can aid with building a desire to engage in adaptive behaviors, such as taking medications as prescribed and following nutrition and exercise plans (63). • Behavioral management strategies can target the behavioral manifestations of distress often seen in young children (64). • Cognitive-behavioral and mindfulness-based therapies can effectively target anxious, irritable, and depressed moods among children and adolescent (65, 66). • Behavioral and cognitive-behavioral therapies can target chronic pain and poor sleep (67, 68). • Psychiatric consultation may be indicated for psychotropic medication management (e.g., when depressive symptoms interfere with engagement in therapy). • Interventions should be mindful of children and families’ socioeconomic status, racial-ethnic background, and other demographic factors (69). • Patients with ongoing needs should be provided recommendations for continued care and/or provided with information on online and community supports.
Family Mental Health Care	• Consider the mental health of caregivers and adjustment within the family system.	• Families of pediatric patients with CHD referred to rehabilitation should be assessed for psy­chosocial needs which may hinder the care of patients, should be provided with support as needed, and should be provided with recommendations for psychological in­terventions if abnormal findings are observed which cannot be addressed within the support model. • Psychological in­terventions should be evidence-based and delivered by licensed mental health professionals trained in specific interventions. • For families engaged in psychological intervention, psychometrically sound assessment tools should be repeated over the course of rehabilitation to ensure ongoing monitoring of symptoms and provide outcome measures of care. • Families with ongoing needs should be provided with information on online and community supports.

A portion of children in cardiac ICUs display delirium, a clinical syndrome with acute disturbance in consciousness and cognition that can fluctuate throughout the day (70). In many ICUs, regular screening for delirium is being implemented. However, it should be noted that patients can screen positive for delirium in cases of hypnotic-related iatrogenic withdrawal (71), the anticholinergic syndrome (72), brain injury, developmental delays, or neuroirritability. Misdiagnoses have significant implications for medical management and outcomes (73). Even though the symptoms of many patients with delirium resolve rapidly (likely because the delirium was a temporary effect of anesthesia or sedation), a portion have persistent symptoms during their intensive care stay.

##### 4.1.6.2. Psychological care

Families of infants with CHD need family-centered care in cardiac ICUs, as the stress of hospitalization can have long-lasting developmental and psychosocial implications (21). Pediatric mental health providers should be readily available in inpatient units, not only to address the distress associated with critical illness and lengthy hospital stays but also to foster healthy behaviors, promote treatment compliance, and help develop self-efficacy (74). This is particularly important for older children and adolescents. Psychological care is further detailed in Table 8.

#### 4.1.7. Discharge planning from acute in-hospital stays

Patients may require additional rehabilitation support upon discharge, depending on the complexity of their cardiac disease and their medical needs, skilled nursing needs, and functional level at discharge. This additional support may include acute inpatient rehabilitation, outpatient interventions, or home-based care.

Inpatient options:

• Discharge to acute inpatient rehabilitation, in which the focus is on the continued delivery of acute medical management, required skilled nursing care, and intensive attention to function. Patients receive three hours of rehabilitation therapy per day.
• Discharge to long-term acute care (LTAC), where the goal is to provide medical management and necessary skilled nursing care. There is no minimum amount of required therapy.

Outpatient options:

• Discharge to early intervention services or school-based therapies, pending qualification for such services.
• Discharge to an outpatient rehabilitation therapy program.
• School-based rehabilitation therapies and accommodations.

### 4.2. Outpatient Rehabilitation Program (Phase II)

Outpatient rehabilitation for children is equivalent to Phase II of Cardiac Rehabilitation in Adults with Acquired Heart Disease. However, since surgery in pediatric patients can occur in infancy or early childhood, outpatient rehabilitation may need to be initiated several years after the initial surgery. This is because certain functional difficulties or disabilities may not be recognized until several years after initial surgery and/or the patients might be too young to participate in certain types of rehabilitation therapy. However, when feasible, interventions should start early and be adapted to the appropriate developmental needs of each CHD patient.

This phase of rehabilitation should include aspects of the more classical cardiac rehabilitation program suggested by the American Heart Association (AHA) (75) and American Association of Cardiovascular and Pulmonary Rehabilitation (AACPVR) (76), such as exercise training, but adapted to meet the unique rehabilitation needs of children and adolescents (as previously described in these recommendations).

#### 4.2.1. Physical and occupational therapy

Physical and occupational therapy are an essential part of the outpatient rehabilitation program. The goals, assessments, and interventions of both physical and occupational therapy are similar in the inpatient and outpatient settings. These have been extensively detailed in our recommendations for inpatient care and would similarly apply to the outpatient setting (Tables 2–4).

#### 4.2.2. Exercise training

Low exercise capacity is a predictor of hospitalization and death for children with CHD (5, 36). A number of studies have demonstrated that exercise training improves peak VO_2_ after CHD surgery in children and adolescents (37, 77–79) and no adverse effects have been noted (Tables 9–11).

**Table 9:** General considerations for exercise training.

Assessment considerations
Review of the electronic medical record
• Cardiac diagnosis • Comorbidities that might increase exercise risk • Medications
Obtain exercise history through chart review and parent/child report
• Document the patient assessment information that reflects the patient's current status and guides the development and implementation of (1) individualized treatment plans based on the patient's unique needs, and (2) a discharge/follow-up plan that reflects progress toward goals and guides long-term plans. • Consider a cardiopulmonary exercise test prior to initiation of the program • Interactively communicate the treatment and follow-up plans with the patient, family and, if feasible, school in collaboration with the primary pediatrician and cardiologist. • In concert with the primary care provider and/or cardiologist, promote medication compliance.

**Table 10:** Exercise training program.

Assessment domain	Assessment considerations	Intervention considerations
Decreased exercise capacity (39, 80–82)	An initial cardiopulmonary exercise test to understand decreased exercise capacity, safety and guide intervention	Currently no consensus on risk stratification: • Budts et al. (83) propose a stratification based on five pillars with clinical and imaging data • FORCE categories based on baseline risk factors and fitness. Individualized exercise prescription that includes aerobic and resistance training based on clinical assessment, diagnostic testing findings and patient goals Exercise prescription should specify frequency (F), intensity (I), time (T), type (T), volume (V) and progression (P) Duration: 12 weeks with insurances covering about 36 sessions Supervised exercise sessions 2–3 times per week ideally Sessions 30–40 min • 60% is aerobic training • 25% resistance training • warm up/cool down Intensity is based on patient risk stratification. • Aerobic is established with Borg scale (12–14 somewhat hard), heart rate reserve and/or the Talk Test. • Resistance Low resistance, high number of repetitions calisthenics, elastic bands or free weights Cardiac telemetry: • initial sessions (for safety reasons and also for patient/family ease) • per protocol and risk stratification Modify program as patient progresses Vital signs (BP, HR, O2 Sat) • at the beginning and end of sessions • if patient becomes symptomatic Sternal precautions per center protocol risk protocol. Often sternal disruption precautions are in place 6–8 weeks post sternotomy and 2 weeks for prone positioning. • Upper extremity exercises can be added after 6 weeks post op if there are sternal precautions • Exercise program should be stopped if (84) - Decrease in ventricular rate with increasing workload associated with extreme fatigue, dizziness, or other symptoms suggestive of insufficient cardiac output - Failure of heart rate to increase with exercise, and extreme fatigue, dizziness, or other symptoms suggestive of insufficient cardiac output - Progressive fall in systolic blood pressure with increasing workload - Severe hypertension, > 250 mm Hg systolic or 125 mm Hg diastolic, or blood pressures higher than can be measured by the laboratory equipment - Dyspnea that the patient finds intolerable - Symptomatic tachycardia that the patient finds intolerable - Progressive fall in oxygen saturation to <90% or a 10-point drop from resting saturation in a patient who is symptomatic - Presence of ≥3 mm flat or downward sloping ST-segment depression - Increasing ventricular ectopy with increasing workload, including a > 3-beat run - Patient requests termination of the program
General CHD Counseling	Assess: • current knowledge of condition and involvement in the patient's self-care of the patient. • Current involvement in the patient's self-care and perception of physical activity of the family • learning style. • barriers to education, e.g., language, health literacy.	• Key components-structured and sequenced curricula; reinforcement; active participation; collaboration; autonomy; feedback; multiple exposures; and, problem-solving. • Afford recognition of a subjective “normal” exercise feeling/response as some patients might have not participated in structured physical activity. • Active participation in medication compliance. • Collaborative nutritional education and weight management • Provide resources for community activity/exercise/sports and encourage the same. • Reinforcement of the home exercise program (HEP) • Ongoing feedback for the above. • Provide resources for mental health support • Provide parent/family guidance and support to be able to promote PA in their child • Partnership with program to promote successful transition to adult congenital care is advised in each center where this is available. • current knowledge of condition and involvement in the patient's self-care of the patient. • Current involvement in the patient's self-care and perception of physical activity of the family
Physical Activity Counseling (85–87)	• Assess current physical activity level (and determine physical goals. • The physical activity questionnaire for older children (PAQ-C) and Adolescents (PAQ-A): Assess overall sedentary and physical activity levels (88). • Physical Activity Enjoyment Scale (PACES): Assesses positive affect associated with involvement in physical activity (89). • Play, Lifestyle & Activity in Youth (PLAY) questionnaire: Assess all physical literacy domains and has separate child and parent sections (90). • 10-item Self-Esteem Scale: assess global self-esteem (90, 91). • Assess barriers to increased physical activity, and social support in making positive changes. Barriers to youth physical activity (BYPA) and facilitators to youth physical activity (FYPA) (92)	• Provide advice, support, and counseling about physical activity needs on initial evaluation and in follow-up. • Target exercise program to meet individual needs. • Provide educational materials as part of counseling efforts. • Encourage patients to participate in 60 min or more per day of moderate-intensity physical activity every day. • Limit screen/sedentary time to <2 h for patients older than 5 years • Encourage participation in structured physical activity at least twice a week. • Advise low-impact aerobic activity with low resistance component • Reassess the patient's ability to perform activities such as exercise training program progresses.

**Table 11:** Precautions and considerations for exercise training in congenital heart disease.

Recommendations/precautions by type of CHD (85)
Certain hereditary cardiomyopathy, long QT syndrome, other congenital channelopathies, and congenital coronary artery anomalies that can cause arrhythmias considered higher risk for arrhythmias. These patients should follow recommendations from the Heart Rhythm Society Guidelines (93)
Patients with these conditions are encouraged to participate in activities with low-moderate dynamic and static component (94): • Coronary artery compression or insufficiency. • Significant pulmonary hypertension • Severe left or right ventricular outflow tract (LVOT/RVOT) obstruction. • Dilation of the aorta—Severe dilation of the aorta is associated with increased risk of aortic dissection. • Anticoagulation: contact sports/high speed impact are not recommended. • ICD/PM contact sports specific guidelines are being created, however contact and high-speed sports are also discouraged.

The goal of outpatient exercise training (both pre- and postoperatively) is to not only improve exercise capacity and physical activity but also provide the patient with tools and support to become as functional and independent as possible and to improve their overall quality of life. Over the past few years, there has been a major effort to develop more structured cardiac rehabilitation/exercise training programs (39, 80, 81) to provide a framework for these types of interventions. However, the effect of structured rehabilitation/exercise training programs on patient outcomes has yet to be demonstrated.

#### 4.2.3. Speech and language therapy

##### 4.2.3.1 Outpatient speech and language assessment

Throughout their childhood, children with CHD often require periodic monitoring of their speech and language development on an outpatient basis (13). Assessments provided by a speech-language pathologist consider the child's age, individual needs and skills, and etiology of deficits (e.g., developmental vs. acquired). Evaluations may target a range of speech, language, pragmatic, and cognitive skills using standardized testing, criterion-referenced measures, and clinical observation to collect diagnostic information (31). Table 5 outlines possible components and considerations for a comprehensive evaluation of speech, language, and related skills in children with CHD.

##### 4.2.3.2 Outpatient speech and language intervention

The typical goal of speech and language intervention is to optimize overall communicative function, thereby supporting social and academic potential, enhancing emotional well-being, reducing frustration, and improving overall quality of life. Treatment frequency and intensity may change over time based on the number and types of skill areas targeted and the consolidation of learned skills (95–98). Outpatient therapies may be supported in community clinics (e.g., hospital or private clinics and centers), early intervention programs, or in schools (in either individual or group settings.) Children less able to use spoken language to support daily communication may also need augmentative and alternative communication strategies. Over time, changes in health status or the need for subsequent cardiac surgery may impact communication skills and/or the type of intervention approach required to maximize benefit. Therefore, ongoing monitoring and multidisciplinary coordination are recommended. Table 5 details outpatient speech assessment and intervention.

#### 4.3. Feeding and swallowing

##### 4.3.1. Outpatient feeding and swallowing evaluation

Evaluation of feeding and swallowing function is based on current skills, age, nutritional needs, and parental preferences/concerns. Those who required assisted feeding preoperatively are at greater risk of being discharged with a feeding tube (51). For infants, assessment typically focuses on bottle feeding and/or breastfeeding, with a comprehensive evaluation of the infant's sucking skills, coordination of the nutritive sucking pattern, and endurance for feeding. Infants who undergo cardiac surgery within the first month of life may demonstrate feeding difficulties that span the first two or more years of life (99) and therefore benefit from subsequent evaluation. Assessments in children focus on a larger variety of drinking delivery methods and food textures to evaluate oral motor skills, swallowing, and sensory-based feeding difficulties. Table 6 further outlines feeding and swallowing assessment methods and considerations for infants and children with CHD (Table 6).

##### 4.3.2. Outpatient feeding and swallowing intervention

Outpatient feeding and swallowing intervention plans are determined based on the evaluation of patients’ skills, areas of need, age, and service availability. Infant feeding treatment typically targets changes in the feeding environment, positioning during feeding, nipple flow rate, or other therapeutic interventions to improve the feeding dynamic. As indicated, treatment may also target oral aversion and feeding difficulties for infants who do not yet accept oral feeding. Therapeutic interventions are recommended when aspiration is observed during the instrumental assessment. When patients do not respond to these interventions, altered liquid consistencies may be trialed with close monitoring and in collaboration with the medical team due to potential gastrointestinal morbidities.

Children may present with feeding difficulties that are multi-factorial, including oral-motor delays, sensory-based difficulties, learned behavioral difficulties, and ongoing or newly acquired aspiration. Longer-term intervention plans may be established to support these needs. Given the sometimes slow and gradual progression of skills and potentially complex difficulties demonstrated by children, caregiver education and counseling are also typically provided (Table 7).

#### 4.4. Psychology and neuropsychology

##### 4.4.1. Psychological and neuropsychological evaluations

Patients with CHD should be screened for developmental, neuropsychological, and psychosocial concerns in the outpatient setting; this can help the rehabilitation team individualize targets and services for each patient. For guidelines on the recommended timing of follow-up evaluations, assessment tools, and special testing considerations, readers are referred to the guidelines for neurodevelopmental follow-up clinics with children with congenital heart defects (13, 18, 19) (Table 8).

##### 4.4.2. Psychological care

Psychologists and neuropsychologists in outpatient rehabilitation programs can provide cognitive rehabilitation designed to teach specific cognitive skills and establish compensatory mechanisms for impaired cognitive domains (Table 8). Psychologists can also help foster healthy behaviors; promote treatment compliance; address emotional distress, disruptive behaviors, and social skills deficits; and support pain management and sleep hygiene. Some patients might benefit from additional evaluation by psychiatry. In addition, psychologists may be involved in mental health interventions for families, who are at risk of traumatic stress and other concerns (100) and whose mental health can impact the beneficial effect of exercise training programs on pediatric patients' quality of life (101).

Psychological care may improve not only psychosocial health but also physical health. In a meta-analysis of 23 randomized controlled trials involving adult cardiac patients, psychological care reduced emotional distress and improved systolic blood pressure, heart rate, and cholesterol levels (101). In a study of a pediatric cardiac rehabilitation program with a stress management component, physiological measurements were similarly improved, although the study did not separate the effects of exercise training, health education, and stress management (67). Research has also found associations between physical and emotional health among youths who have completed cardiac rehabilitation (102), and between emotional health and daily physical activity among children with CHDs (38).

## 5. Discussion

Rehabilitation needs in pediatric patients with CHD are very different from those of adults with acquired heart disease. As such, rehabilitation programs for patients with CHD should be designed to support the array of functional difficulties described here, and this article provides a valuable framework for developing such programs. Given the lack of available literature and data on pediatric cardiac rehabilitation, we developed this framework based on expert recommendations regarding best practices. The individuals contributing to this article respectively have expertise in rehabilitation, cardiology, cardiac surgery, and neuropsychology, all with a specific focus on our target pediatric population. Our framework addresses rehabilitation in both the inpatient and outpatient settings and incorporates roles for a multidisciplinary pediatric cardiac rehabilitation team. Further research is needed to quantify the impact of these multidisciplinary interventions and help us continue to tailor these programs to the specific needs of the pediatric cardiac population.
